# Establishing the pig as a large animal model for vaccine development against human cancer

**DOI:** 10.3389/fgene.2015.00286

**Published:** 2015-09-15

**Authors:** Nana H. Overgaard, Thomas M. Frøsig, Simon Welner, Michael Rasmussen, Mette Ilsøe, Maria R. Sørensen, Mads H. Andersen, Søren Buus, Gregers Jungersen

**Affiliations:** ^1^Department of Immunology and Vaccinology, National Veterinary Institute, Technical University of DenmarkCopenhagen, Denmark; ^2^Department of International Health, Immunology and Microbiology, University of CopenhagenCopenhagen, Denmark; ^3^Center for Cancer Immune Therapy, Department of Hematology, Copenhagen University HospitalHerlev, Denmark

**Keywords:** immune therapy, cancer vaccines, cytotoxic T cells, animal model, peptide-MHC stability, adjuvants, immunologic

## Abstract

Immunotherapy has increased overall survival of metastatic cancer patients, and cancer antigens are promising vaccine targets. To fulfill the promise, appropriate tailoring of the vaccine formulations to mount *in vivo* cytotoxic T cell (CTL) responses toward co-delivered cancer antigens is essential. Previous development of therapeutic cancer vaccines has largely been based on studies in mice, and the majority of these candidate vaccines failed to induce therapeutic responses in the subsequent human clinical trials. Given that antigen dose and vaccine volume in pigs are translatable to humans and the porcine immunome is closer related to the human counterpart, we here introduce pigs as a supplementary large animal model for human cancer vaccine development. IDO and RhoC, both important in human cancer development and progression, were used as vaccine targets and 12 pigs were immunized with overlapping 20mer peptides spanning the entire porcine IDO and RhoC sequences formulated in CTL-inducing adjuvants: CAF09, CASAC, Montanide ISA 51 VG, or PBS. Taking advantage of recombinant swine MHC class I molecules (SLAs), the peptide-SLA complex stability was measured for 198 IDO- or RhoC-derived 9-11mer peptides predicted to bind to SLA-1^*^04:01, −1^*^07:02, −2^*^04:01, −2^*^05:02, and/or −3^*^04:01. This identified 89 stable (t½ ≥ 0.5 h) peptide-SLA complexes. By IFN-γ release in PBMC cultures we monitored the vaccine-induced peptide-specific CTL responses, and found responses to both IDO- and RhoC-derived peptides across all groups with no adjuvant being superior. These findings support the further use of pigs as a large animal model for vaccine development against human cancer.

## Introduction

Therapeutic anti-cancer vaccines are expected to be important in the future immunotherapeutic treatment of cancer, either alone or in combination with, e.g., administration of drugs targeting the checkpoint inhibitors cytotoxic T-lymphocyte-associated protein 4 (CTLA-4) and programmed cell death 1 (PD-1) (Hodi et al., 2010; Brahmer et al., 2012; Topalian et al., 2012; Hamid et al., 2013; Wolchok et al., 2013). Previously, vaccines have mainly been comprised of full proteins; however especially in terms of safety, peptide-based vaccines are preferable, as reviewed in Purcell et al. (2007). Many cancer-associated targets have been described (Cheever et al., 2009; Andersen et al., 2012), and peptide vaccinations have previously generated significant immune responses, although these only rarely correlated with the clinical outcome (Dalgleish and Whelan, 2006; Becker et al., 2012; Inderberg-Suso et al., 2012). One limitation to peptide vaccination is that peptides in general generate weaker responses *in vivo* as compared to full protein, and the immunogenicity of peptides alone is not sufficient to generate a strong immune response; therefore adjuvant systems are included to enhance such response in order to increase the likelihood of a clinical effect. Numerous adjuvant systems have proved the ability to strongly stimulate the unspecific immune system in a cancer setting (Butterfield, 2015) hence supporting the need for studies identifying optimal Th_1_-inducing adjuvants in combination with cancer antigens.

Various mice models are currently the golden standard for early pre-clinical studies, even though important differences in terms of immunology and physiology between mice and humans exist. It is now well established that “mice lie” and recent studies have shown the pig immunome to be much more similar to the human counterpart (Dawson et al., 2013; Seok et al., 2013); pigs must therefore be considered a highly relevant supplementary model when studying human immune activation. Furthermore, the difference in body size and metabolism makes studies on the dose effect of adjuvants and peptides impossible to extrapolate from mice studies to human vaccine formulation. Relating specifically to cancer, six genetic defects are required for converting both normal porcine and human cells to their cancerous counterparts (Hahn et al., 1999; Adam et al., 2007), while only two mutations are required to convert a mouse cell (Rangarajan et al., 2004). Over the last decade the toolbox of swine immunological reagents has expanded considerably, which contributes further to the usefulness of pigs as a relevant model for human diseases (Meurens et al., 2012). In this study we introduce outbred pigs as a large animal model for human cancer vaccine development.

The immune response to cancer is complex and responses can either be in favor or disfavor of cancer development and progression. Major players in the anti-cancer immune response are the CD8^+^ cytotoxic T lymphocytes (CTLs), which specifically recognize peptides derived from cancer-specific or over-expressed proteins when presented by the Major Histocompatibility Complex (MHC) class I molecules on the surface of the transformed cells or cross-presented by dendritic cells. Mounting strong and effective CTL responses against such peptide-MHC complexes is thus a goal of vaccine development against cancer. Previous work has shown indoleamine 2,3-dioxygenase (IDO) and Ras homolog gene family member C (RhoC) to be promising antigen targets for inclusion in vaccines against multiple cancer forms (Wenandy et al., 2008; Sørensen et al., 2009). To investigate whether it is possible to mount immune responses toward the above mentioned cancer antigens, we immunized 12 healthy outbred pigs holding the swine leukocyte antigen (SLA)-1^*^04:01, SLA-3^*^04:01, SLA-1^*^07:02, and/or SLA-2^*^05:02 MHC class I alleles with 20mer overlapping peptides spanning the entire sequence of IDO and RhoC. The pigs were divided in four adjuvant groups receiving the 20mer peptide library formulated in either poly(I:C) decorated dimethyldioctadecylammonium (DDA)/monomycoloyl glycerol (MMG) cationic liposomes referred to as the cationic adjuvant formulation (CAF)09 (Korsholm et al., 2014), a porcine/human modification of the combined adjuvant for synergistic activation of cellular immunity (CASAC) containing CpG, monophosphoryl lipid A (MPL), IFN-γ, CD40 ligand (CD40L), and CD40L enhancer in an oil/water formulation (Wells et al., 2008), Montanide ISA 51 VG water/oil (Iversen et al., 2014) (hereafter referred to as ISA 51 VG) or phosphate buffered saline (PBS). These adjuvants were chosen based on their previous ability to mount CTL responses in mouse and/or man. CAF09 has shown promising results in a mouse tumor model, where it generated responses to multiple antigens in parallel (Korsholm et al., 2014) and has previously been used several times in pigs (data not published). CASAC has shown very promising results in mice, where it generated high numbers of antigen-specific CD8^+^ T cells (Wells et al., 2008). Among numerous human cancer studies, ISA 51 VG has together with a short IDO-derived peptide been shown to induce clinical responses in human metastatic lung cancer patients (Iversen et al., 2014) and has been used in various other clinical trials for cancer treatment (Tsuji et al., 2013; Lennerz et al., 2014).

In order to compare the vaccine-induced antigen-specific CD8^+^ CTL response toward IDO and RhoC between the adjuvant groups, we predicted 198 9-11mer ligands by use of the NetMHCcons prediction server (Karosiene et al., 2012) in combination with the Position Scanning Combinatorial Peptide Library (PSCPL) method (as exemplified in Pedersen et al., 2011). Out of these, a total of 89 stable peptide-MHC complexes were subsequently identified using *in vitro* stability measurements. Pigs were blood sampled at various time points before and after immunizations and the IFN-γ responses following ~70 h of peripheral blood mononuclear cell (PBMC)-peptide co-culture suggested generation of CTL responses to cancer antigens following peptide immunization. All adjuvants were capable of generating some CTL responses although none of the adjuvants was found to be superior. Taken together this first vaccine trial supports the use of pigs as a large animal model for human anti-cancer vaccine development. Stronger and more consistent responses are, however, warranted indicating the relevance of further studies on adjuvant and peptide dose, number of immunizations and more detailed characterization of the immunological response profile.

## Materials and methods

### Animals

Outbred Danish Landrace/Yorkshire/Duroc pigs were obtained from a Danish production farm (Askelygaard, Roskilde, Denmark). Upon arrival to the National Veterinary Institute, the pigs were housed in groups of six animals using straw as bedding material with water freely available and food supplied once a day. No additional environmental enrichment was provided. All procedures of animal handling and experimentation were internally and externally approved by the institutional committee and the Danish Animal Experiments' Inspectorate, respectively.

### Blood sampling

Blood samples from pigs were collected at day −35, −9, −2, 12, 33, 40, and day 54. The PBMCs were isolated using Lymphoprep gradient separation in SepMate tubes (both from Stemcell Technologies, Grenoble, France) from blood samples obtained at day −2, 12, 33, 40, and 54 and used directly in the IFN-γ release assay.

### SLA-typing of candidate pigs

Five weeks old, non-sex matched pigs were blood sampled and SLA-typed prior to purchase. Sanger sequencing based SLA-typing: Genomic DNA was extracted from blood samples obtained at day -35 using DNeasy® Blood & Tissue Kit (Qiagen, Cat. No. 69504) according to the manufacturer's instructions. PCR with sequence specific primers and subsequent sequencing of the positive amplicons (by Eurofins, Ebersberg, Germany) allowed for detection of the alleles SLA-1^*^04:01, SLA-1^*^07:02, SLA-2^*^04:01, SLA-2^*^05:02, and SLA-3^*^04:01 as previously described (Pedersen et al., 2014b).

NGS-based SLA-typing and Expression Analysis: To confirm the presence of the SLA class I genes found by the previously described SLA-typing, we used next generation sequencing of PCR amplicons spanning exon 2 and 3 of SLA class I genes, which also allowed for expression analysis of the transcripts. RNA from blood samples obtained at day -9 was purified using PAXgene Blood RNA Kit (PreAnalytiX, Cat. No. 762174) according to the manufacturer's instructions. After enzymatic digestion of genomic DNA, the RNA was transcribed into cDNA using the QuantiTect® Reverse Transcription Kit (Qiagen, Cat.No.205311). The cDNA was used as a template in a PCR with barcoded primers designed in conserved areas of the exon 2 and 3 of all known SLA class I genes. After sequencing on the MiSeq™ 250PE platform (The National High-throughput DNA Sequencing Centre, University of Copenhagen, Denmark) the sequences were de-multiplexed, pair mate joined, quality checked and sorted into clusters showing the expression levels of each allele (Ilsøe et al., manuscript in preparation). This was followed by alignment against a library containing all previously described SLA class I alleles to determine the allele identity.

### 20mer overlapping peptide library for immunization

Fifty-nine 20mer peptides with 10mer overlap covering the entire IDO and RhoC amino acid (aa) sequence were purchased from Genscript (New Jersey, U.S) or Pepscan Presto BV (Lelystad, the Netherlands). Due to dissolving problems, peptide IDO_330−350_ was omitted and only 58 peptides were included in the immunization protocol (**Table 2**). Peptides were dissolved to a concentration of 5 mM in milliQ water, N-methyl-2-pyrrolidone or 3% ammonia water in accordance with the supplier's recommendations, for further details see Supplementary Table 1.

### Immunizations

Based on their SLA profile 12 animals (12 weeks old) were divided in four groups each containing three pigs, hence maximizing the MHC class I allelic coverage in each group. The animals were primed at day 0 and boosted at day 19 with the full 20mer overlapping peptide library in combination with either an adjuvant system or PBS. Each pig received 50 μg for priming and 25 μg for boosting of each peptide with the exception of certain peptides (Supplementary Table 1). The CAF09 adjuvant (Korsholm et al., 2014) was a generous gift from Dennis Christensen at the State Serum Institute, Copenhagen, Denmark and vaccine doses for this group of animals were formulated by gentle mixing of 1 ml peptide library diluted in 10 mM Tris buffer with 1 ml CAF09. A porcine/human modification of CASAC (Wells et al., 2008) was prepared with the MegaCD40L® [1 μg recombinant human CD154 and 2 μg CD154 enhancer (Enzo Life Sciences, NY, U.S.)], 500 μg CpG ODN2007 (ODN 2007 Class B CpG oligonucleotide—bovine/porcine TLR9 ligand, InvivoGen, CA, U.S.) and 1 μg recombinant porcine IFN-γ (R&D Systems, UK) all formulated in PBS and mixed with peptides in a total volume of 1 ml followed by gentle mixing with 1 ml of Sigma adjuvant (Sigma Adjuvant System, Sigma Aldrich, Missouri, U.S.). Montanide ISA 51 VG (Seppic, Puteaux, France) vaccines were prepared by thorough mixing of 1 ml peptide library formulated in PBS and 1 ml adjuvant through an i-connector according to manufacturer's instructions. Finally, a vaccine formulated in PBS was produced with suspension of the peptide library in PBS only. All vaccines were formulated in a total volume of 2 ml and administered subcutaneously into the flank except for ISA 51 which was administered intramuscularly. Priming and boosting were both administered into the left side of the animals. Pigs were monitored for 5 days following priming and boosting, and all animals remained healthy following both injections.

### 9-11mer peptide library for immune monitoring

Porcine IDO and RhoC aa sequences were obtained from the Uniprot database (http://www.uniprot.org/uniprot/F6K2E8 and http://www.uniprot.org/uniprot/F2Z5K4). Using PSCPL (Stryhn et al., 1996) and the NetMHCcons1.1 server (Karosiene et al., 2012) we identified 198 9-11mer peptides predicted to bind to at least one of the five in-house SLAs (SLA-1^*^04:01, SLA-1^*^07:02, SLA-2^*^04:01, SLA-2^*^05:02, and SLA-3^*^04:01) with a rank score ≤ 2 % by at least one of the prediction methods. These were synthesized via Fmoc-based chemistry and purchased from Pepscan Presto BV (Lelystad, the Netherlands). For further information see Supplementary Table 2. These peptides were referred to as IDO1-IDO136 and RhoC1-RhoC62.

### Test of peptide-MHC complex stability

The stability of the selected 198 peptides in complex with relevant SLA class I molecules and β_2_m was determined using a scintillation proximity assay (SPA), as previously described (Harndahl et al., 2011). Briefly, biotinylated recombinant MHC class I heavy chains were attached to a streptavidin-coated scintillation microplate together with iodinated (^125^I) β_2_m and candidate peptide resulting in a scintillation signal, which was consecutively measured every 40 min by a scintillation plate counter. The duration of this signal is directly correlated to the stability of the peptide-MHC class I-β_2_m complex under dissociating conditions and in the presence of excess unlabeled β_2_m (Harndahl et al., 2011). Peptides with a half-life ≥ 0.5 h were selected as stable binders.

### *In vitro* peptide stimulation of porcine PBMCs

In a 96 well plate, 2 × 10^5^ PBMCs/well were individually cultured with 5 μg/ml of each of the 80 peptides previously found to form a total of 89 stable complexes with SLA class I heavy chain and β_2_m. Cells co-cultured with Staphylococcal Enterotoxin B (SEB) (1 μg/ml) and media alone (RPMI 1640 (Gibco, Life Technologies) supplemented with 10 % fetal calf serum) were used as positive and negative controls, respectively. Cells were cultured for 67.5–70.0 h and the supernatant was harvested, frozen at −20°C and subsequently analyzed for IFN-γ release by an ELISA method.

### IFN-γ release assay

Quantification of IFN-γ in the supernatant from cells stimulated with 9-11mer peptides was carried out in a monoclonal ELISA as previously described (Riber et al., 2011) except that the plates were developed for 1–30 min with tetramethylbenzidine (Kem-En-Tec, Taastrup, Denmark) at RT. The absorbance at 450 nm was determined using a microplate reader (Thermo Scientific) and corrected for unspecific background by subtraction of the signal at 650 nm. The detection limit was established as 8.8 pg/ml and all measurements below this limit was set at 8.8 pg/ml for further calculations. A vaccine-induced response was defined as the increase from pre- to post-vaccination after subtraction of the background IFN-γ from media control cultures without added peptides. Samples from three pigs (numbers 2033, 2045, and 2107) were excluded from the analysis as negative control media cultures had high non-specific background with IFN-γ levels above 20 pg/ml. All positive control SEB cultures were above the 70 pg/ml cut-off.

### Statistics

Due to the low number of animals, statistical analyses between different adjuvants are not meaningful and no statistical analyses to prove significant differences were attempted.

## Results

**Figure 1:**
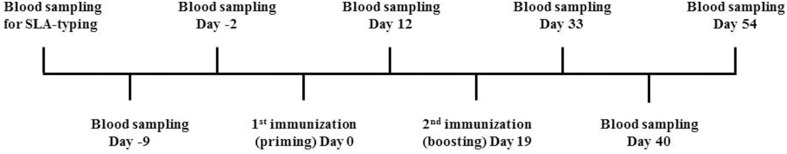
**Overview of the immunization strategy**. Prior to initiation of the vaccine trial, candidate pigs were blood sampled and SLA-typed in order to select animals holding one or more of the following SLA alleles: SLA-1^*^04:01, SLA-1^*^07:02, SLA-2^*^04:01, SLA-2^*^05:02, and SLA-3^*^04:01. Pigs were then purchased and blood sampled at day −2 to determine the background level of IFN-γ. At day 0, pigs were primed with 58 20mer overlapping peptides in combination with either CAF09, CASAC, ISA 51VG or PBS. Blood samples were obtained at day 12, and all pigs were then boosted with another round of immunization at day 19. Blood samples were obtained three times following boost namely at day 33, 40, and 54.

Previous studies have confirmed the involvement of CD8^+^ T cells in anti-cancer immune reactivity (Klebanoff et al., 2005; Sørensen et al., 2011b; Andersen, 2012; Joyce and Fearon, 2015; Rosenberg and Restifo, 2015) and anti-cancer vaccines are generally administered with the aim of enhancing this antigen- specific T-cell reactivity. To establish the pig as a large animal model for human cancer vaccine development, we constructed a monitoring platform for vaccine-induced T-cell reactivity. First, candidate pigs were blood sampled and SLA-typed in order to choose animals holding the relevant SLA class I molecules (Figure 1). Second, identification of proteins relevant for vaccination and prediction of candidate CD8^+^ T cell epitopes from the full protein sequence were carried out. The *in vitro* stability of the candidate T cell epitopes in complex with relevant SLA class I molecule was then examined. Pigs were immunized with 20mer overlapping peptides and blood sampled at various time points pre- and post-immunization in order to monitor the T-cell reactivity *ex vivo* (Figure 1). To increase the knowledge obtained from this study, we stratified the pigs in four groups based on their SLA-profile and immunized each group with peptides in combination with an adjuvant system or PBS.

### SLA class I typing

We obtained blood from 24 animals and probed them for the presence of SLA-1^*^04:01, SLA-2^*^04:01, SLA-1^*^07:02, SLA-2^*^05:02, and SLA-3^*^04:01 by Sanger sequencing (Table 1 and data not shown) corresponding to our in-house recombinant SLA library. Twelve pigs were positive for at least one of the desired alleles and further NGS-based SLA-typing, which also included expression analysis on RNA samples from these animals, confirmed part of these along with the identification of a few more expressed SLA molecules (Table 1). SLA-3^*^04:01 was found in nine of the pigs (75%) and seven pigs (58%) showed expression of SLA-1^*^04:01 (Table 1). SLA-1^*^07:02 was found in 50% of the animals, whereas SLA-2^*^05:02 and SLA-2^*^04:01 were found in only one (8%) and none of the pigs, respectively (Table 1).

**Table 1:** **SLA-profile of pigs included in the immunization trial**.

**Adjuvant**	**Pig**	SLA-1^*^04:01	SLA-1^*^07:02	SLA-2^*^05:02	SLA-3^*^04:01
CAF09	2107				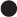
	2035	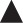	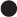		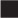
	2038		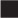		
CASAC	2034	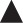			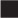
	2037	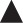	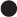		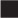
	2042		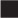		
ISA 51 VG	2046	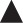	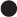		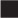
	2041	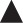		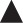	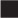
	2043	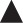			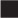
PBS	2033				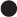
	2045	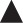			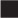
	2040		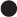		

*SLA-typing of animals included in the immunization trial. Animals were blood sampled and tested for the presence of SLA-1^*^04:01, SLA-1^*07:^02, SLA-2^*^04:01, SLA-2^*^05:02, and SLA-3^*^04:01 at both the DNA and mRNA level. 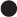, determined by Sanger sequencing-based SLA-typing; 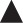, determined by NGS-based SLA-typing and expression analysis; 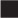, determined by both methods. None of the animals were positive for SLA-2^*^04:01 hence this molecule was left out of the table. The Sanger sequencing-based SLA-typing was unsuccessful for SLA-1^*^04:01*.

### Analysis of peptide-MHC class I binding stability

**Figure 2:**
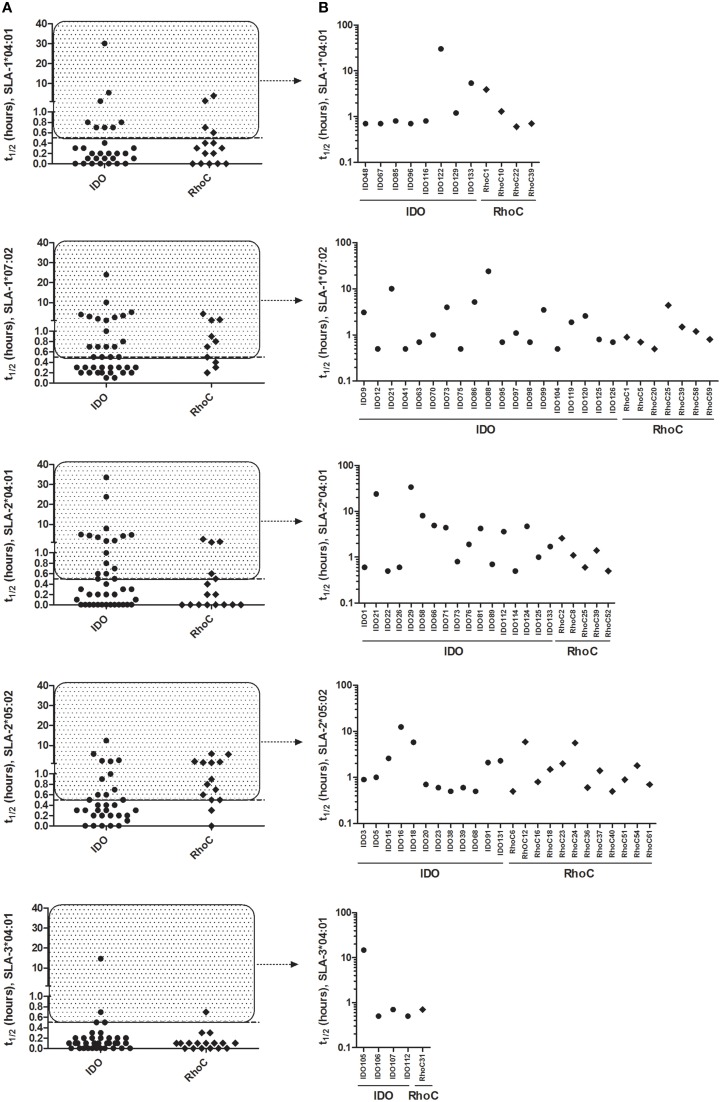
**Peptide-MHC class I binding stability as determined by SPA analysis**. Stability of the predicted peptides in the 9-11mer peptide library with relevant SLA molecules measured by the Scintillation Proximity Assay. **(A)** SPA-determined half-life of peptides predicted as binders for each SLA molecule. **(B)** Individual peptides with SPA-determined half-life ≥0.5 h (stably binding) for each SLA molecule. Peptides derived from IDO and RhoC are shown in circles and diamonds, respectively.

In order to determine peptides capable of forming stable (t_1∕2_≥0.5 h) complexes with the relevant SLA amongst the 198 peptides, the half-life of the peptide-MHC class I binding was determined for each of the 244 predicted peptide-SLA complexes using SPA analysis. For the widely distributed SLA-1^*^04:01, a total of 12 IDO and RhoC-derived peptides formed stable complexes with this SLA molecule (Figure 2A), and especially IDO122 showed very high binding stability (t_1∕2_ = 30.2 h, Figure 2B). Also, 26 peptides formed stable complexes with the other well-distributed SLA-type amongst the pigs, namely SLA-1^*^07:02 (Figure 2A). Here, especially IDO21 (t_1∕2_ = 10.1 h) and IDO88 (t_1∕2_ = 24.0 h) were found to form highly stable complexes (Figure 2B). Strikingly, only five peptides were found to form stable complexes with SLA-3^*^04:01 in the SPA analysis (Figure 2A), and IDO105 was the only peptide being able to form a complex with a half-life longer than 1 h with this SLA molecule (t_1∕2_ = 14.7 h) (Figure 2B). A total of 24 peptides were shown to form stable complexes with SLA-2^*^05:02 (Figure 2A) with IDO16 (t_1∕2_ = 12.5 h) forming the most stable complex. IDO21 and IDO29 were both found to form stable complexes with SLA-2^*^04:01 exhibiting half-lives of 23.8 and 33.6 h, respectively (Figure 2B), and additional 20 peptides also formed stable complexes with SLA-2^*^04:01. To sum up, the SPA analysis revealed 89 stable peptide-SLA complexes (80 different peptides) from the 244 predicted high-affinity complexes (198 different peptides), and these were used in subsequent analyses of the CD8^+^ T-cell reactivity.

### IFN-γ responses

**Figure 3:**
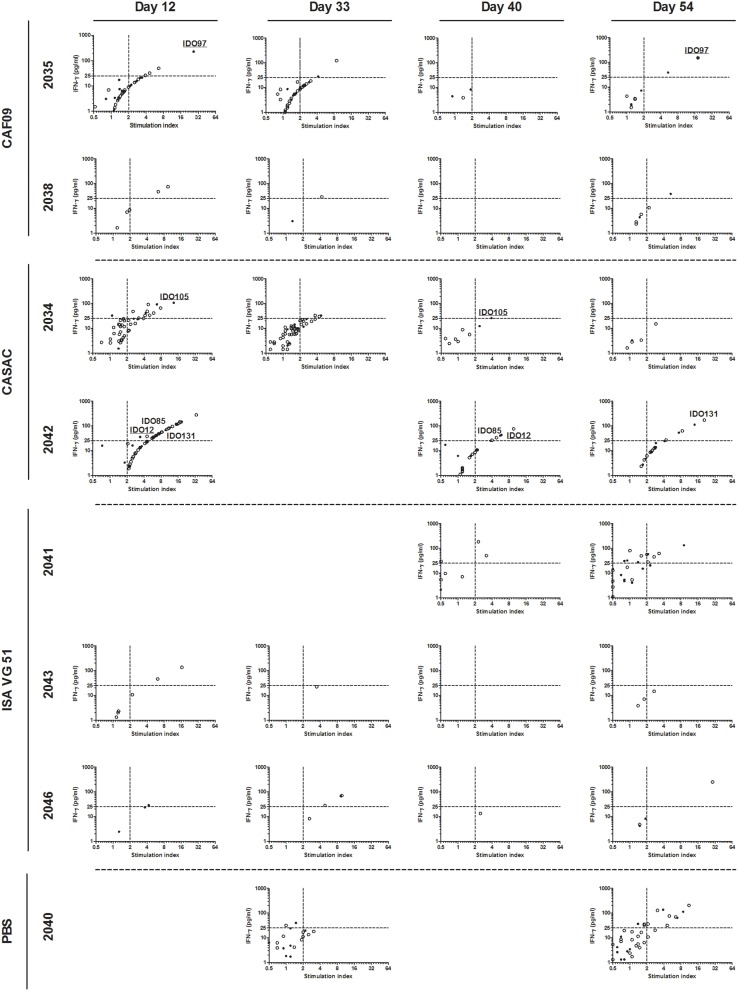
**IFN-γ responses to the stable binders in the 9-11mer peptide library**. PBMCs purified from immunized pigs at day −2, 12, 33, 40, and 54 were stimulated with 80 peptides found to be stably binding to one or more of SLA-1^*^04:01, SLA-1^*^07:02, SLA-2^*^04:01, SLA-2^*^05:02, and SLA-3^*^04:01. A biological relevant vaccine-induced response was defined as a 2-fold increase as compared to day −2 (dashed line, x-axis) and a concentration of IFN-γ equaling at least 25 pg/ml (dashed line, y-axis). For each animal, peptides were divided into two groups: stable (filled circles) and non-stable (open circles) referring to the binding stability of a given peptide correlated with the SLA-profile of each pig. Responses occurring repeatedly in individual animals are highlighted by peptide name with stably binding peptides being underlined. Animals with no responses at any time point, and time points for included animals with data not fulfilling the quality parameters were left out of the analyses.

**Figure 4:**
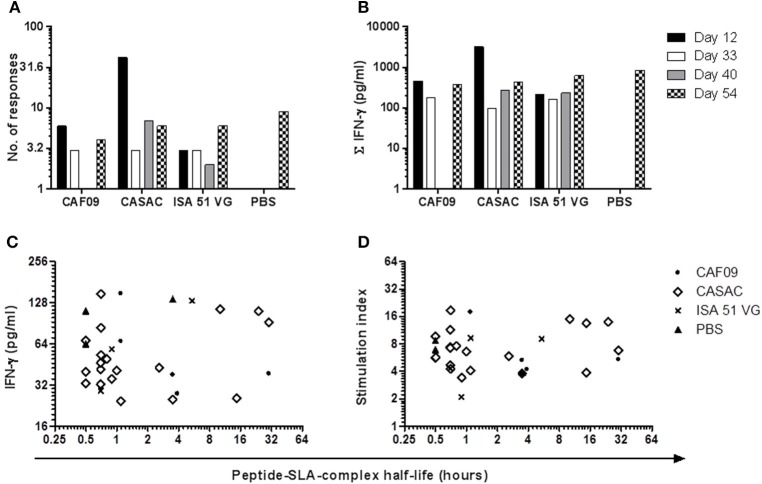
**Comparison of the adjuvant systems used in the immunization trial**. The three Th1-inducing adjuvants CAF09, CASAC, and ISA 51 VG were compared on the basis of their ability to generate responses **(A)** and the total amount of peptide-specific IFN-γ produced from the culture **(B)** at the various time points following immunization. For peptides found to be stably binding to MHC class I in the SPA analysis, half-life of the peptide-MHC class I complex was correlated to the amount of IFN-γ **(C)** and the stimulation index **(D)**.

To monitor the induction of specific T cell populations, co-cultures with peptide and PBMCs obtained at different time points prior to and after the immunizations were analyzed for IFN-γ release after ~70 h. From the vaccine-induced responses, a biologically relevant IFN-γ response following peptide co-culture was defined as a 2-fold increase (stimulation index = 2) as compared to pre-immunization (day -2) and with a concentration of 25 pg/ml or more as depicted by the threshold lines (Figure 2). Three pigs were excluded from the analysis due to high non-specific background. For each animal, the peptide responses were divided into two groups based on the measured ability to form stable complexes with the SLA molecules found in the SPA analysis (Table 1, Figure 2). In general, we found responses in all pigs to both stable and non-stable binders (Figure 3, Table 2, Supplementary Table 2). Despite similar SLA profiles the pigs did not respond to the same peptides (Table 2). When comparing the total number of responses of all animals per adjuvant group, CASAC was shown to be slightly superior especially at day 12 after the first immunization; however in general the adjuvant systems performed similarly (Figure 4A). Comparison of the average IFN-γ production (total amount of IFN-γ divided by total number of responses) also revealed that the adjuvants induced a similar level of this cytokine (Figure 4B). Since the stability obtained in the SPA analysis has been used as a determinant for selecting the ligands most likely to be CD8^+^ T cell epitopes, comparisons of the peptide-MHC class I half-lives and IFN-γ production (Figure 4C) as well as stimulation index (Figure 4D) were carried out. Surprisingly, no significant correlations could be drawn from this analysis. More data points are needed, especially for complexes with long half-lives, to fully determine this, but our findings apparently do not support the idea of stably binding peptides being more immunogenic as shown by Harndahl et al. (2012). It should be noted, though, that this study was performed with viral T cell epitopes. As shown in Supplementary Table 2 we find responses against the vast majority of stably binding peptides, however, for a significant part of these there was a mismatch between the predicted SLA restriction and the observed responses.

**Table 2:** **IFN-γ responses following co-culture with the 9-11mer peptide library**.

**SLA molecule**	**Pig**	**Adjuvant**	**Total number of responses**				**Stable binders**			
			**Day 12**	**Day 33**	**Day 40**	**Day 54**	**Day 12**	**Day 33**	**Day 40**	**Day 54**
1^*^04:01 3^*^04:01	**2034**	CASAC	11	3	1	0	3(27.2%)	1(33.3%)	1(100.0%)	–
	**2043**	ISA 51 VG	2	0	0	0	0(0.0%)	–	–	–
1^*^07:02	**2038**	CAF09	2	1	0	1	0(0.0%)	0(0.0%)	–	1(100.0%)
	**2042**	CASAC	31	–	6	6	9(29.0%)	–	3(50.0%)	3(50.0%)
	**2040**	PBS	–	0	–	9	–	–	–	3(33.3%)
1^*^04:01 1^*^07:02 3^*^04:01	**2035**	CAF09	4	2	0	3	1(25%)	1(50.0%)	–	2(66.6%)
	**2046**	ISA 51 VG	1	3	0	0	1(100.0%)	1(33.3%)	–	–
1^*^04:01 2^*^05:02 3^*^04:01	**2041**	ISA 51 VG	–	–	2	6	–	–	0(0.0%)	2(33.3%)

*Summarized responses from PBMCs co-cultured with the 9-11mer peptide library. Pigs are divided into four groups based on their SLA-profile. Adjuvant indicates immunization strategy, i.e., CAF09, CASAC, ISA 51 VG, or PBS injected animals. The total number of responses at day 12, 33, 40, and 54 are shown (column 4–7) as well as the number of responses generated from co-culture with a peptide shown to stably bind the given SLA molecule (column 8–11). The percentage of responses generated from these stable binders is shown in brackets for each time point. Animals with no responses at any time point are left out of the analysis. “–“ indicates that the quality control thresholds of the assay were not fulfilled and therefore these data were left out of the analyses*.

## Discussion

To measure antigen-specific CD8^+^ T cell responses, knowledge of the MHC class I alleles present in each individual is necessary. SLA-3^*^04:01 was found to be the most widely distributed of the five in-house MHC class I alleles; however the SLA-3^*^04:01 molecule has previously been shown to be unstable, most likely due to the presence of only one anchor position (Pedersen et al. manuscript in preparation), and NGS analysis revealed a significantly lower SLA-3^*^04:01 expression level compared to the expression of the SLA-1 and SLA-2 molecules (data not shown). The pigs were divided into the adjuvant groups based on their SLA-profile to stratify the study as much as possible and provide a similar possibility of measuring antigen-specific T-cell responses in all adjuvant groups.

To identify MHC class I ligands, we predicted binders (9-11mer peptides) from the full length sequences of IDO and RhoC to SLA-1^*^04:01, SLA-1^*^07:02, SLA-2^*^04:01, SLA-2^*^05:02, and SLA-3^*^04:01 by the combined use of NetMHCcons and PSCPL. We selected peptides in the upper 2 % rank by either of the methods. This ranking is a measure of the possibility that a random peptide would be a better binder to the relevant MHC complex. The resulting 136 and 62 predicted binders from IDO and RhoC, respectively, were purchased and investigated further (Supplementary Table 1). Some of the peptides ranked ≤ 2 % on more than one allele. The immunogenicity of a peptide has previously been linked to the affinity of its binding to MHC class I (Sette et al., 1994). However, it has recently been suggested that the stability of the peptide-MHC class I binding is a more accurate measure of the peptide's immunogenicity (Harndahl et al., 2012). Along with this note, the NetMHCstab server has recently been established (Jørgensen et al., 2014), but unfortunately this predictor does so far only work for a few human MHC class I molecules. Importantly, it has not previously been possible to measure the peptide-MHC class I stability in a high-throughput manner; however after development of the SPA analysis, an essentially label-free stability screening approach is now possible (Harndahl et al., 2011).

Vaccine efficacy can be determined in various ways, and a typical approach to evaluate Th1-inducing adjuvant systems is to measure the IFN-γ production. Importantly, the level of secreted IFN-γ has been used to evaluate the efficacy of the only FDA-approved therapeutic anti-cancer vaccine, PROVENGE® (sipuleucel-T), in a peptide-immunization study in mice (Saif et al., 2014). Several cell types have the ability to produce IFN-γ including Natural Killer T cells, Natural Killer cells, CD4^+^ and CD8^+^ T cells amongst others. Here, *in vitro* peptide co-culture of PBMCs purified pre- or post-immunization was done with *9-11mer* peptides. Therefore the IFN-γ production observed following this co-culture is expected to originate from *CD8*^+^*T cells* encountering antigen-presenting cells presenting peptides in the context of MHC class I. Since all animals were found to respond to both stable and non-stable peptides as determined by the SPA analysis, it could be indicative of peptides with lower half-life than 0.5 h also having immunogenic potential. Surprisingly, the animals generally did not respond consistently to the same peptides over time. This might however partly be due to blood sampling only showing a snapshot of what is circulating in the blood stream. Also, most pigs, like humans, have three MHC class I gene loci constitutively expressed although duplication of the SLA-1 allele has been observed in a fraction of animals (Lunney et al., 2004). Therefore, most pigs express up to six different MHC class I molecules. We have selectively tested for the five SLAs in our in-house library; however the majority of the animals are expected to express additional SLA molecules and we most likely do not know their full SLA-profile. The additional SLA class I molecules might also bind certain peptides in the 9-11mer library, which could account for some of the responses to peptides not shown to bind stably to our in-house SLA molecules.

In the majority of the animals, especially pigs 2034, 2035, 2040, and 2042, there seemed to be a correlation between the stimulation index (as compared to day −2, pre-immunization) and the amount of peptide-specific IFN-γ produced. Unfortunately we had to exclude three pigs, two from the PBS and one from the CAF09 group, from these analyses due to high non-specific background. Recruitment of pigs from a background of higher sanitation status could provide a cleaner response window for future studies. The animal receiving peptides formulated in PBS only also showed responses to IDO and RhoC at day 54 post-immunization; however naturally occurring CTL responses to endogenous cancer antigens, among them IDO, have been frequently observed in healthy humans as well (Visseren et al., 1995; Jäger et al., 2001; Sørensen et al., 2011a; Frøsig et al., 2015). Further of notice, two of the previous peptide vaccine human trials with the most convincing clinical data used the granulocyte-macrophage colony stimulating factor as the only adjuvant (Inderberg-Suso et al., 2012; Walter et al., 2012). This cytokine functions as a growth factor and does not stimulate immunity directly as more common adjuvants do. Still, however, it seems the identification of an optimal adjuvant system will be highly beneficial.

Following immunization we found responses in most animals and for most time points; albeit the secreted amount of IFN-γ was fairly low, and the response magnitude can possibly be enhanced by further optimizing the immunization protocol. The generation of only a few specific T cells could, however, be enough to induce epitope spreading generating mutation antigen-specific T cells of higher avidity in a human cancer setting. The frequency of specific T cell populations measured in previous peptide vaccination studies in human cancer patients have also generally been low (Pollack et al., 2014; Køllgaard et al., 2015). This is probably due to the endogenous origin of the targets, but nonetheless, in some of these studies a clear clinical effect has been obtained. This was the situation in the IDO peptide immunization trial (Iversen et al., 2014) where 47 % of the treated patients developed a long-lasting partial response or stable disease, defined as 8.5 months compared to the expected 6–7 months of progression-free survival in this patient group. In addition, treated patients had significantly longer overall survival than a cohort of patients of similar shape not treated with the vaccine. Immunological activity was found *ex vivo* in this trial, but at quite low frequencies. In contrast, high-frequency immune responses are often found in cancer immunization studies using murine models (Wei et al., 2015) as exemplified in the study by Zhao et al. (2012), where more than 1000 pg/ml IFN-γ was secreted for most peptides tested after two immunizations and only 48 h of peptide stimulation *ex vivo*. For comparison, the maximum level of secreted IFN-γ observed in our trial was 280 pg/ml IFN-γ (pig 2042, day 12, peptide IDO15, Figure 3) following 72 h of stimulation.

Here, we included three different Th1-inducing adjuvants, namely CAF09, CASAC, and ISA 51 VG and immunized three pigs with each along with a fourth group receiving peptides in PBS only. The PBS group was included as a control for the adjuvant efficacy. From this study, none of the adjuvants were found to be superior as they were all capable of generating a CTL response toward cancer antigens found to be important in human disease. Thus, despite very convincing data from mouse studies (Wells et al., 2008), this first porcine/human modification of CASAC was not superior in the pig model. The inconsistent response profile highlights, however, the importance of further optimization of peptide immunization protocols. In this study we set out to analyze the vaccine-induced immune reactivity on fresh material. Since the SPA analysis resulted in a peptide library of 80 stably binding peptides, a total of approximately 1000 stimulations and 2000 ELISA well analyses were carried out for each time point to measure the secreted IFN-γ. Due to this large screening setup, analyzing more cytokines in parallel was not feasible. To confirm the induction of antigen-specific CD8^+^ T cells following an immunization protocol, MHC multimer staining for flow cytometry analysis is usually performed in humans and mice. Although we have developed porcine MHC multimers and staining protocols (Pedersen et al., 2014a), we are still in the process of developing a high-throughput MHC multimer screening system for porcine cells. We believe the pig model is highly appropriate to address questions relating to optimal adjuvant composition and formulation, peptide repertoire and dosing, as well as the route and number of administrations for endogenous peptide immunizations. Supplemented with the porcine immunological reagents, including recombinant SLA class I molecules and SLA multimers, we will further be able to characterize the immunological response profile following different immunization protocols and relate this to the immunological correlates of anti-cancer protection.

## Author contributions

NO and TF designed and performed the experiments, analyzed the data and wrote the article. GJ conceived the approach, designed the experiments, approved the analysis, provided financial support and co-wrote the article. SW, MR, MI, designed and performed experiments. MS, MR, and SW analyzed the data. SB and MA supervised the study.

## Funding

This study was supported by The Danish Council for Independent Research for Technology and Production (ID: DFF-4005-00428).

### Conflict of interest statement

The authors declare that the research was conducted in the absence of any commercial or financial relationships that could be construed as a potential conflict of interest.

## Abbreviations

CAF09: Cationic Adjuvant Formulation 09
CASAC: Combined Adjuvant for Synergistic Activation of Cellular Immunity
CTL: Cytotoxic T cell
IDO, Indoleamine 2: 3-dioxygenase
ISA 51 VG: Montanide ISA 51 VG
MHC: Major Histocompatibility Complex
PBS: Phosphate Buffered Saline
PBMC: Peripheral Blood Mononuclear Cell
RhoC: Ras homolog gene family member C
SEB: Staphylococcal Enterotoxin B
SLA: Swine Leukocyte Antigen
SPA: Scintillation Proximity Assay.
