# Supplementary material for: Establishing the pig as a large animal model for vaccine development against human cancer
Source: Front Genet. 2015 Sep 15;6:286. doi: 10.3389/fgene.2015.00286 (PMC4584933; doi:10.3389/fgene.2015.00286)
Supplement: Supplementary file 1 [file Table1.PDF]

**Supplementary material for Overgaard et al. 2015, Frontiers in Genetics**

**Supplementary Table 1. 20-mer overlapping immunization library**

| Antigen | Sequence | Position | Amount (µg) | Dissolving agent |
|---|---|---|---|---|
| **IDO** | MALDWWSPMDNSWKIFEEYH | $IDO_{1-20}$ | ≥50/25 | W |
| **IDO** | NSWKIFEEYHIDEDLGFALP | $IDO_{10-30}$ | | A |
| **IDO** | IDEDLGFALPNPLEELPHPY | $IDO_{20-40}$ | | W |
| **IDO** | NPLEELPHPYDAWIAIAKNL | $IDO_{30-50}$ | | A |
| **IDO** | DAWIAIAKNLPELIKNGQLR | $IDO_{40-60}$ | | W |
| **IDO** | PELIKNGQLRAEVEKLATLS | $IDO_{50-70}$ | | W |
| **IDO** | AEVEKLATLSIDGLQGHKMQ | $IDO_{60-80}$ | | W |
| **IDO** | IDGLQGHKMQRLAHLVLGYI | $IDO_{70-90}$ | | W |
| **IDO** | RLAHLVLGYITMAYVWGQGD | $IDO_{80-100}$ | | N |
| **IDO** | TMAYVWGQGDEDIRKVLPRN | $IDO_{90-110}$ | | W |
| **IDO** | EDIRKVLPRNIAIPYCKLSE | $IDO_{100-120}$ | | W |
| **IDO** | IAIPYCKLSEKLGLPPILVY | $IDO_{110-130}$ | 40/20 | W |
| **IDO** | KLGLPPILVYADCVLANWKK | $IDO_{120-140}$ | | W |
| **IDO** | ADCVLANWKKKDPSGPMTYK | $IDO_{130-150}$ | | W |
| **IDO** | KDPSGPMTYKNMDILFSFPG | $IDO_{140-160}$ | | W |
| **IDO** | NMDILFSFPGGDCGKGFFLV | $IDO_{150-170}$ | ≥50/25 | N |
| **IDO** | GDCGKGFFLVSLLVEIAAAS | $IDO_{160-180}$ | 34/17 | N |
| **IDO** | SLLVEIAAASAIKVIPTLFN | $IDO_{170-190}$ | | A |
| **IDO** | AIKVIPTLFNAIQCEDLDAL | $IDO_{180-200}$ | | N |
| **IDO** | AIQCEDLDALQKALLDITSS | $IDO_{190-210}$ | | W |
| **IDO** | QKALLDITSSLHKALEVFHQ | $IDO_{200-220}$ | 37/18.5 | W |
| **IDO** | LHKALEVFHQIHEYVDPKLF | $IDO_{210-230}$ | | W |
| **IDO** | IHEYVDPKLFFNVLRIYLSG | $IDO_{220-240}$ | ≥50/25 | W |
| **IDO** | FNVLRIYLSGWKGNPLLSEG | $IDO_{230-250}$ | | N |
| **IDO** | WKGNPLLSEGLLYEGVWDTP | $IDO_{240-260}$ | | A |
| **IDO** | LLYEGVWDTPKKFAGGSAAQ | $IDO_{250-270}$ | | W |
| **IDO** | KKFAGGSAAQSSIFQCFDVL | $IDO_{260-280}$ | | W |
| **IDO** | SSIFQCFDVLLGVQHTVGGV | $IDO_{270-290}$ | 34/17 | W |
| **IDO** | LGVQHTVGGVPSGSAAGFLQ | $IDO_{280-300}$ | | W |
| **IDO** | PSGSAAGFLQEMRTYMPPAH | $IDO_{290-310}$ | | W |
| **IDO** | EMRTYMPPAHRNFLHSLESG | $IDO_{300-320}$ | | W |
| **IDO** | RNFLHSLESGPSVREFVLSK | $IDO_{310-330}$ | | W |
| **IDO** | PSVREFVLSKGDALLQETYN | $IDO_{320-340}$ | 28/14 | A |
| **IDO** | GDALLQETYNECVQAMVSLR | $IDO_{330-350}$ | - | N |
| **IDO** | ECVQAMVSLRNYHLQIVTKY | $IDO_{340-360}$ | ≥50/25 | N |
| **IDO** | NYHLQIVTKYIVIPASQQAK | $IDO_{350-370}$ | | W |
| **IDO** | IVIPASQQAKKKQASEEPSE | $IDO_{360-380}$ | | W |
| **IDO** | KKQASEEPSEEENRGTGGTN | $IDO_{370-390}$ | | W |
| **IDO** | EENRGTGGTNVIDFLKTVRG | $IDO_{380-400}$ | | W |
| **IDO** | VIDFLKTVRGTTVRSLLKEG | $IDO_{390-410}$ | 45/22.5 | W |

| Antigen | Sequence | Position | Amount (µg) | Dissolving agent |
|---|---|---|---|---|
| **RhoC** | VGDGACGKTCLLIVFSKDQF | $RhoC_{10-30}$ | | A |
| **RhoC** | LLIVFSKDQFPEVYVPTVFE | $RhoC_{20-40}$ | | A |
| **RhoC** | PEVYVPTVFENYIADIEVDG | $RhoC_{30-50}$ | 28/14 | A |
| **RhoC** | NYIADIEVDGKQVELALWDT | $RhoC_{40-60}$ | | A |
| **RhoC** | KQVELALWDTAGQEDYDRLR | $RhoC_{50-70}$ | | A |
| **RhoC** | AGQEDYDRLRPLSYPDTDVI | $RhoC_{60-80}$ | 48/24 | W |
| **RhoC** | PLSYPDTDVILMCFSIDSPD | $RhoC_{70-90}$ | | W |
| **RhoC** | LMCFSIDSPDSLENIPEKWT | $RhoC_{80-100}$ | | N |
| **RhoC** | SLENIPEKWTPEVKHFCPNV | $RhoC_{90-110}$ | 30/15 | W |
| **RhoC** | PEVKHFCPNVPIILVGNKKD | $RhoC_{100-120}$ | | W |
| **RhoC** | PIILVGNKKDLRQDEHTRRE | $RhoC_{110-130}$ | | W |
| **RhoC** | LRQDEHTRRELAKMKQEPVR | $RhoC_{120-140}$ | | W |
| **RhoC** | LAKMKQEPVRSEEGRDMANR | $RhoC_{130-150}$ | | W |
| **RhoC** | SEEGRDMANRISAFGYLECS | $RhoC_{140-160}$ | 33/16.5 | N |
| **RhoC** | ISAFGYLECSAKTKEGVREV | $RhoC_{150-170}$ | | W |
| **RhoC** | AKTKEGVREVFEMATRAGLQ | $RhoC_{160-180}$ | | W |
| **RhoC** | FEMATRAGLQVRKNKRRRGC | $RhoC_{170-190}$ | | W |
| **RhoC** | VRKNKRRRGCPIL | $RhoC_{180-193}$ | 43/21.5 | W |

The 20-mer IDO- or RhoC-derived peptides with 10-mer overlap used for the immunization. The peptide position indicates the position relative to the first amino acid. Peptides were dissolved in 3% ammonium water (A), N-methyl-2-pyrrolidone (N) or milliQ water (W). Unless otherwise stated, 50µg of each peptide were used for priming and 25µg for boosting. Due to difficulties dissolving $IDO_{330-350}$, this peptide was left out of the immunization protocol.
