# Supplementary material for: Establishing the pig as a large animal model for vaccine development against human cancer
Source: Front Genet. 2015 Sep 15;6:286. doi: 10.3389/fgene.2015.00286 (PMC4584933; doi:10.3389/fgene.2015.00286)
Supplement: Supplementary file 2 [file Table2.PDF]

Supplementary material for Overgaard et al. 2015, Frontiers in Genetics

Supplementary Table 2. The 9-11-mer peptide library

Peptide	Sequence	Position	Length	SLA restriction	Binder ($t_{1/2}$ ≥ 0.5 h)	T cell response
IDO1	AGFLQEMRTY	$IDO_{296\text{-}205}$	10-mer	2*04:01	0.6	x
IDO2	AIAKNLPEL	$IDO_{45\text{-}53}$	9-mer	1*04:01	-	
IDO3	AIAKNLPELI	$IDO_{45\text{-}54}$	10-mer	2*05:02	0.9	
IDO4	ALEVFHQIHEY	$IDO_{214\text{-}224}$	11-mer	1*04:01	-	
IDO5	ALQKALLDI	$IDO_{199\text{-}207}$	9-mer	2*05:02	-	x
IDO6	ANWKKKDPS	$IDO_{136\text{-}144}$	9-mer	3*04:01	-	
IDO7	AQSSIFQCF	$IDO_{269\text{-}277}$	9-mer	1*04:01	N/A	
IDO8	ASAIKVIPTLF	$IDO_{179\text{-}189}$	11-mer	1*04:01<br>2*04:01	-<br>-	
IDO9	CVQAMVSLRNY	$IDO_{342\text{-}352}$	11-mer	1*04:01<br>1*07:02	-<br>3.1	
IDO10	DIRKVLPRNI	$IDO_{102\text{-}111}$	10-mer	2*05:02	-	
IDO11	DNSWKIFEEY	$IDO_{10\text{-}19}$	10-mer	2*04:01	-	
IDO12	DPSGPMTYKNM	$IDO_{142\text{-}152}$	11-mer	1*07:02	0.5	x
IDO13	DWWSPMDNSW	$IDO_{4\text{-}13}$	10-mer	2*04:01	-	
IDO14	EELPHPYDAWI	$IDO_{34\text{-}44}$	11-mer	2*05:02	-	
IDO15	EIAAASAIKV	$IDO_{175\text{-}184}$	10-mer	2*05:02	2.6	x
IDO16	EIAAASAIKVI	$IDO_{175\text{-}185}$	11-mer	1*04:01<br>2*05:02	0.8<br>12.5	x
IDO17	EKLGLPPILVY	$IDO_{120\text{-}130}$	11-mer	2*04:01	-	
IDO18	ELPHPYDAWI	$IDO_{35\text{-}44}$	10-mer	2*05:02	5.8	
IDO19	ENRGTGGTNVI	$IDO_{382\text{-}392}$	11-mer	2*05:02	-	
IDO20	EVEKLATLSI	$IDO_{62\text{-}71}$	10-mer	2*05:02	0.7	x
IDO21	EVFHQIHEY	$IDO_{216\text{-}224}$	9-mer	1*07:02<br>2*04:01	10.1<br>23.8	x
IDO22	FALPNPLEEL	$IDO_{27\text{-}36}$	10-mer	2*04:01	0.5	x
IDO23	FLHSLESGPSV	$IDO_{313\text{-}323}$	11-mer	2*05:02	0.6	x
IDO24	FLQEMRTYM	$IDO_{298\text{-}306}$	9-mer	1*07:02	-	
IDO25	FLVSLLVEI	$IDO_{168\text{-}176}$	9-mer	2*05:02	-	
IDO26	FNAIQCEDL	$IDO_{189\text{-}197}$	9-mer	2*04:01	0.6	x
IDO27	FNVLRIYLS	$IDO_{231\text{-}239}$	9-mer	3*04:01	-	
IDO28	FNVLRIYLSG	$IDO_{231\text{-}240}$	10-mer	3*04:01	-	
IDO29	FNVLRIYLSGW	$IDO_{231\text{-}241}$	11-mer	2*04:01	33.6	x
IDO30	FPGGDCGKGFF	$IDO_{158\text{-}168}$	11-mer	1*07:02	-	
IDO31	GGDCGKGFF	$IDO_{160\text{-}168}$	9-mer	1*04:01	N/A	
IDO32	GPSVREFVL	$IDO_{320\text{-}328}$	9-mer	1*07:02	N/A	
IDO33	GSAAQSSIF	$IDO_{266\text{-}274}$	9-mer	1*04:01<br>2*04:01	-<br>-	
IDO34	GVPSGSAAGF	$IDO_{289\text{-}298}$	10-mer	1*04:01	-	
IDO35	HIDEDLGFAL	$IDO_{20\text{-}29}$	10-mer	1*04:01	-	

Peptide	Sequence	Position	Length	SLA restriction	Binder ($t_{1/2} \geq 0.5$ h)	T cell response
IDO36	HKALEVFHQI	$IDO_{212-221}$	10-mer	2*05:02	-	
IDO37	HKMQRLAHLVL	IDO_{77-87}	11-mer	3*04:01	-	
IDO38	HLQIVTKYI	$IDO_{353-362}$	9-mer	2*05:02	0.5	
IDO39	HLQIVTKYIVI	$IDO_{353-363}$	11-mer	2*05:02	0.6	
IDO40	HLVLGYITM	IDO_{84-92}	9-mer	1*07:02	-	
IDO41	HLVLGYITMAY	IDO_{84-94}	11-mer	1*07:02	0.5	
IDO42	HPYDAWIAI	IDO_{38-46}	9-mer	1*07:02	-	
IDO43	HPYDAWIAIAK	IDO_{38-48}	11-mer	1*07:02	-	
IDO44	IAIAKNLPELI	IDO_{44-54}	11-mer	2*05:02	-	
IDO45	KALEVFHQI	$IDO_{213-221}$	9-mer	2*05:02	-	
IDO46	KALLDITSSL	$IDO_{202-211}$	10-mer	3*04:01	-	
IDO47	KDPSGPMTY	$IDO_{141-149}$	9-mer	3*04:01	-	
IDO48	KGDALLQETY	$IDO_{330-339}$	10-mer	1*04:01<br>2*04:01	0.7<br>-	x
IDO49	KGFFLVSLL	$IDO_{165-173}$	9-mer	2*05:02<br>3*04:01	-<br>-	
IDO50	KGFFLVSLLV	$IDO_{165-174}$	10-mer	3*04:01	-	
IDO51	KKDPSGPMTY	$IDO_{140-149}$	10-mer	1*04:01	-	
IDO52	KLFFNVLRI	$IDO_{228-236}$	9-mer	3*04:01	-	
IDO53	KLFFNVLRIY	$IDO_{228-237}$	10-mer	1*07:02<br>3*04:01	-<br>-	
IDO54	KLFFNVLRIYL	$IDO_{228-238}$	11-mer	3*04:01	-	
IDO55	KLGLPPILVY	$IDO_{121-130}$	10-mer	1*07:02	-	
IDO56	KLSEKLGLPPI	$IDO_{117-127}$	11-mer	2*05:02	-	
IDO57	KMQRLAHLVL	IDO_{78-87}	10-mer	3*04:01	-	
IDO58	KNMDILFSF	$IDO_{150-158}$	9-mer	2*04:01<br>3*04:01	8.0<br>-	x
IDO59	KNMDILFSFPG	$IDO_{150-160}$	11-mer	3*04:01	-	
IDO60	KTVRGTTVRSL	$IDO_{396-406}$	11-mer	3*04:01	-	
IDO61	KVIPTLFNAI	$IDO_{183-192}$	10-mer	2*05:02	-	
IDO62	KVLPRNIAI	$IDO_{105-113}$	9-mer	2*05:02<br>3*04:01	-<br>-	
IDO63	KVLPRNIAIPY	$IDO_{105-115}$	11-mer	1*04:01<br>1*07:02<br>3*04:01	-<br>0.7<br>-	x
IDO64	LDWWSPMDNSW	IDO_{3-13}	11-mer	2*04:01	-	
IDO65	LGLPPILVY	$IDO_{122-130}$	9-mer	1*07:02<br>2*04:01	-<br>-	
IDO66	LGYITMAYVW	IDO_{87-96}	10-mer	2*04:01	4.9	x
IDO67	LLDITSSLH	$IDO_{204-212}$	9-mer	1*04:01	0.7	x
IDO68	LLVEIAAASAI	$IDO_{172-182}$	11-mer	2*05:02	0.5	x
IDO69	LPNPLEELPH	IDO_{29-38}	10-mer	1*07:02	-	
IDO70	LPRNIAIPY	$IDO_{107-115}$	9-mer	1*07:02	1.0	x
IDO71	LSEGLLYEGVW	$IDO_{247-257}$	11-mer	2*04:01	4.4	x

Peptide	Sequence	Position	Length	SLA restriction	Binder ($t_{1/2}$ ≥ 0.5 h)	T cell response
IDO72	LSEKLGLPPI	$IDO_{118-127}$	10-mer	2*05:02	-	
IDO73	LVLGYITMAY	IDO_{85-94}	10-mer	1*07:02<br>2*04:01	4.0<br>0.8	
IDO74	LVYADCVLANW	$IDO_{128-138}$	11-mer	1*04:01<br>2*04:01	N/A<br>-	
IDO75	MALDWWSPM	IDO_{1-9}	9-mer	1*07:02<br>3*04:01	0.5<br>-	x
IDO76	MDNSWKIFEEY	IDO_{9-19}	11-mer	2*04:01	1.9	x
IDO77	MPPAHRNFLH	$IDO_{306-315}$	10-mer	1*07:02	-	
IDO78	MQRLAHLVL	IDO_{79-87}	9-mer	3*04:01	-	
IDO79	MQRLAHLVLGY	IDO_{79-89}	11-mer	1*07:02<br>2*04:01<br>3*04:01	-<br>-<br>-	
IDO80	MTYKNMDIL	$IDO_{147-155}$	9-mer	2*04:01<br>2*05:02<br>3*04:01	-<br>-<br>-	
IDO81	MTYKNMDILF	$IDO_{147-156}$	10-mer	1*04:01<br>1*07:02<br>2*04:01<br>2*05:02<br>3*04:01	-<br>-<br>4.2<br>-<br>-	
IDO82	MTYKNMDILFS	$IDO_{147-157}$	11-mer	3*04:01	-	
IDO83	MVSLRNYHL	$IDO_{346-354}$	9-mer	1*04:01	-	
IDO84	MVSLRNYHLQI	$IDO_{346-356}$	11-mer	2*05:02	-	
IDO85	NMDILFSFP	$IDO_{152-159}$	9-mer	1*04:01	0.8	x
IDO86	NPLEELPHPY	IDO_{31-40}	10-mer	1*07:02	5.2	
IDO87	NPLLSEGLL	$IDO_{244-252}$	9-mer	1*07:02	N/A	
IDO88	NPLLSEGLLY	$IDO_{244-253}$	10-mer	1*07:02	24.0	x
IDO89	NSWKIFEEY	IDO_{11-19}	9-mer	1*07:02<br>2*04:01	-<br>0.7	
IDO90	NSWKIFEEYH	IDO_{11-20}	10-mer	2*04:01	-	
IDO91	NSWKIFEEYHI	IDO_{11-21}	11-mer	2*05:02	2.1	x
IDO92	NVLRIYLSGW	$IDO_{232-241}$	10-mer	2*04:01	N/A	
IDO93	NYHLQIVTKY	$IDO_{351-360}$	10-mer	2*04:01	-	
IDO94	PLEELPHPY	IDO_{32-40}	9-mer	1*04:01	-	
IDO95	PLLSEGLLY	$IDO_{245-253}$	9-mer	1*07:02	0.7	
IDO96	PMDNSWKIF	IDO_{8-16}	9-mer	1*04:01	0.7	
IDO97	PPAHRNFLH	$IDO_{307-315}$	9-mer	1*07:02	1.1	x
IDO98	PPAHRNFLHSL	$IDO_{307-317}$	11-mer	1*07:02	0.7	x
IDO99	PPILVYADCV	$IDO_{125-134}$	10-mer	1*07:02	3.5	x
IDO100	QAMVSLRNY	$IDO_{344-352}$	9-mer	1*07:02	-	
IDO101	QEMRTYMPP	$IDO_{300-308}$	9-mer	3*04:01	-	
IDO102	QRLAHLVLGY	IDO_{80-89}	10-mer	2*04:01<br>3*04:01	-<br>-	

Peptide	Sequence	Position	Length	SLA restriction	Binder ($t_{1/2}$ ≥ 0.5 h)	T cell response
IDO103	RGTGGTNVIDF	$IDO_{384-394}$	11-mer	1*04:01	-	
IDO104	RLAHLVLGY	IDO_{81-89}	9-mer	1*04:01<br>1*07:02<br>2*04:01	-<br>0.5<br>-	
IDO105	RNFLHSLES	$IDO_{311-319}$	9-mer	3*04:01	14.7	x
IDO106	RNFLHSLESG	$IDO_{311-320}$	10-mer	3*04:01	0.5	
IDO107	RNFLHSLESGP	$IDO_{311-321}$	11-mer	3*04:01	0.7	x
IDO108	RNIAIPYCKL	$IDO_{109-118}$	10-mer	3*04:01	-	
IDO109	RNIAIPYCKLS	$IDO_{109-119}$	11-mer	3*04:01	-	
IDO110	RNYHLQIVT	$IDO_{350-358}$	9-mer	2*04:01<br>3*04:01	-<br>-	
IDO111	RNYHLQIVTK	$IDO_{350-359}$	10-mer	2*04:01<br>3*04:01	-<br>-	
IDO112	RNYHLQIVTKY	$IDO_{350-360}$	11-mer	2*04:01<br>3*04:01	3.6<br>0.5	
IDO113	RTYMPPAHRNF	$IDO_{303-313}$	11-mer	1*04:01<br>2*04:01<br>2*05:02<br>3*04:01	1.0<br>-<br>-<br>-	N/A
IDO114	SAIKVIPTLF	$IDO_{180-189}$	10-mer	2*04:01<br>2*05:02	0.5<br>-	x
IDO115	SIDGLQGHKM	IDO_{70-79}	10-mer	1*04:01	-	
IDO116	SLESGPSVREF	$IDO_{316-326}$	11-mer	1*04:01	0.8	x
IDO117	SLHKALEVF	$IDO_{210-218}$	9-mer	1*04:01<br>1*07:02	-<br>-	
IDO118	SLRNYHLQI	$IDO_{348-356}$	9-mer	2*05:02<br>3*04:01	-<br>-	
IDO119	SPMDNSWKI	IDO_{7-15}	9-mer	1*07:02	1.9	x
IDO120	SPMDNSWKIF	IDO_{7-16}	10-mer	1*07:02	2.6	x
IDO121	SSLHKALEVF	$IDO_{209-218}$	10-mer	2*04:01	-	
IDO122	SAAQSSIFQCF	$IDO_{267-277}$	11-mer	1*04:01<br>2*04:01	30.2<br>-	x
IDO123	TSSLHKALEVF	$IDO_{208-218}$	11-mer	1*04:01<br>2*04:01	-<br>-	
IDO124	TYMPPAHRNF	$IDO_{304-313}$	10-mer	2*04:01	4.7	x
IDO125	VLGYITMAY	IDO_{86-94}	9-mer	1*04:01<br>1*07:02<br>2*04:01	-<br>1.0<br>0.8	
IDO126	VLPRNIAIPY	$IDO_{106-115}$	10-mer	1*04:01<br>1*07:02	-<br>0.7	x
IDO127	VLSKGDALL	$IDO_{327-335}$	9-mer	2*05:02	-	
IDO128	VPSGSAAGF	$IDO_{290-298}$	9-mer	1*07:02<br>2*04:01	-<br>-	
IDO129	VQAMVSLRNY	$IDO_{343-352}$	10-mer	1*04:01<br>2*04:01	1.2<br>-	x

Peptide	Sequence	Position	Length	SLA restriction	Binder ($t_{1/2}$ ≥ 0.5 h)	T cell response
IDO130	VSLRNYHLQI	$IDO_{347-356}$	10-mer	2*05:02<br>3*04:01	-<br>-	
IDO131	WKIFEEYHI	IDO_{13-21}	9-mer	2*05:02	2.3	x
IDO132	WWSPMDNSW	IDO_{5-13}	9-mer	2*04:01	-	
IDO133	YADCVLANW	$IDO_{130-138}$	9-mer	1*04:01<br>2*04:01	5.4<br>1.7	x
IDO134	YHLQIVTKY	$IDO_{352-360}$	9-mer	1*07:02<br>2*04:01	-<br>-	
IDO135	YVDPKLFFNV	$IDO_{224-233}$	10-mer	1*04:01	-	
IDO136	YVDPKLFFNVL	$IDO_{224-234}$	11-mer	1*04:01	-	
RhoC1	ALWDTAGQEDY	$RhoC_{54-64}$	11-mer	1*04:01<br>1*07:02	3.9<br>0.9	x
RhoC2	ANRISAFGY	$RhoC_{148-156}$	9-mer	2*04:01<br>3*04:01	2.6<br>-	x
RhoC3	ANRISAFGYL	$RhoC_{148-157}$	10-mer	3*04:01	-	
RhoC4	ANRISAFGYLE	$RhoC_{148-158}$	11-mer	3*04:01	-	
RhoC5	CPNVPIILV	$RhoC_{107-115}$	9-mer	1*07:02	0.7	x
RhoC6	DLRQDEHTR	$RhoC_{120-128}$	9-mer	2*05:02	0.5	x
RhoC7	DMANRISAF	$RhoC_{146-154}$	9-mer	1*04:01		
RhoC8	DMANRISAFGY	$RhoC_{146-156}$	11-mer	1*04:01<br>1*07:02<br>2*04:01	N/A<br>-<br>1.1	x
RhoC9	DSLENIPEKW	$RhoC_{90-99}$	10-mer	2*04:01	-	
RhoC10	DTDVILMCF	$RhoC_{76-84}$	9-mer	1*04:01	1.3	
RhoC11	DTDVILMCFSI	$RhoC_{76-86}$	11-mer	2*05:02	N/A	
RhoC12	DVILMCFSI	$RhoC_{78-86}$	9-mer	2*05:02	5.9	x
RhoC13	DYDRLRPLSY	$RhoC_{65-74}$	10-mer	1*04:01	-	
RhoC14	EDYDRLRPLSY	$RhoC_{64-74}$	11-mer	1*04:01<br>2*04:01	-<br>-	
RhoC15	ELAKMKQEP	$RhoC_{130-138}$	9-mer	2*05:02	-	
RhoC16	ELAKMKQEPVR	$RhoC_{130-140}$	11-mer	2*05:02	0.8	x
RhoC17	ENIPEKWTP	$RhoC_{93-101}$	9-mer	2*05:02	1.1	N/A
RhoC18	ENIPEKWTPEV	$RhoC_{93-103}$	11-mer	2*05:02	1.5	x
RhoC19	ENYIADIEVD	$RhoC_{40-19}$	10-mer	2*04:01	-	
RhoC20	EPVRSEEGRDM	$RhoC_{137-147}$	11-mer	1*07:02	0.5	

Peptide	Sequence	Position	Length	SLA restriction	Binder ($t_{1/2}$ ≥ 0.5 h)	T cell response
RhoC21	EVDGKQVEL	$RhoC_{47\text{-}55}$	9-mer	1*04:01	-	
RhoC22	EVDGKQVELAL	$RhoC_{47\text{-}57}$	11-mer	1*04:01	0.6	
RhoC23	EVKHFCPNV	$RhoC_{102\text{-}110}$	9-mer	2*05:02	2.0	x
RhoC24	EVKHFCPNVPI	$RhoC_{102\text{-}112}$	11-mer	2*05:02	5.6	x
RhoC25	EVYVPTVFENY	$RhoC_{32\text{-}42}$	11-mer	1*07:02<br>2*04:01	4.4<br>0.6	
RhoC26	FSKDQFPEVY	$RhoC_{25\text{-}34}$	10-mer	2*04:01	-	
RhoC27	KEGVREVFEM	$RhoC_{164\text{-}173}$	10-mer	3*04:01	-	
RhoC28	KHFCPNVPI	$RhoC_{104\text{-}112}$	10-mer	3*04:01	-	
RhoC29	KHFCPNVPII	$RhoC_{104\text{-}113}$	11-mer	3*04:01	-	
RhoC30	KHFCPNVPIIL	$RhoC_{104\text{-}114}$	11-mer	3*04:01	-	
RhoC31	KNKRRRGCPI	$RhoC_{183\text{-}192}$	10-mer	3*04:01	0.7	x
RhoC32	KNKRRRGCPIL	$RhoC_{183\text{-}193}$	11-mer	3*04:01	-	
RhoC33	KRRRGCPIL	$RhoC_{185\text{-}193}$	9-mer	3*04:01	-	
RhoC34	KTKEGVREVF	$RhoC_{162\text{-}171}$	10-mer	3*04:01	-	
RhoC35	LSYPDTDVI	$RhoC_{72\text{-}80}$	9-mer	2*04:01<br>2*05:02	-<br>-	
RhoC36	LSYPDTDVIL	$RhoC_{72\text{-}81}$	10-mer	2*05:02	0.6	x
RhoC37	LSYPDTDVILM	$RhoC_{72\text{-}82}$	11-mer	2*04:01<br>2*05:02	-<br>1.4	
RhoC38	LWDTAGQEDY	$RhoC_{57\text{-}66}$	10-mer	1*04:01	-	
RhoC39	MANRISAFGY	$RhoC_{147\text{-}156}$	10-mer	1*04:01<br>1*07:02<br>2*04:01	0.7<br>1.5<br>1.4	x
RhoC40	MAAIRKKLVI	$RhoC_{1\text{-}10}$	10-mer	2*05:02	0.5	
RhoC41	NKRRRGCPI	$RhoC_{184\text{-}192}$	9-mer	3*04:01	-	
RhoC42	RDMANRISAF	$RhoC_{145\text{-}154}$	10-mer	1*04:01<br>2*04:01<br>3*04:01	-<br>-<br>-	
RhoC43	RISAFGYLE	$RhoC_{150\text{-}158}$	9-mer	1*04:01	-	
RhoC44	RKNKRRRGCPI	$RhoC_{182\text{-}192}$	11-mer	3*04:01	-	
RhoC45	RLRPLSYPD	$RhoC_{68\text{-}76}$	9-mer	3*04:01	-	

Peptide	Sequence	Position	Length	SLA restriction	Binder ($t_{1/2} \geq 0.5$ h)	T cell response
RhoC46	RPLSYPDTDV	$RhoC_{70-79}$	10-mer	1*07:02	-	
RhoC47	SIDSPDSLENI	$RhoC_{85-95}$	11-mer	1*04:01	-	
RhoC48	SKDQFPEVY	$RhoC_{26-34}$	9-mer	1*04:01<br>2*04:01<br>2*05:02	-<br>-<br>N/A	
RhoC49	SLENIPEKW	$RhoC_{91-99}$	9-mer	1*04:01<br>2*04:01	-<br>-	
RhoC50	SLENIPEKWTP	$RhoC_{91-101}$	11-mer	1*04:01	-	
RhoC51	TVFENYIADI	$RhoC_{37-46}$	10-mer	2*05:02	0.9	x
RhoC52	VDGKQVELALW	$RhoC_{48-58}$	11-mer	2*04:01	0.5	x
RhoC53	VFSKDQFPEVY	$RhoC_{24-34}$	11-mer	2*04:01	-	
RhoC54	VPTVFENYI	$RhoC_{35-43}$	9-mer	2*05:02	1.8	
RhoC55	WDTAGQEDY	$RhoC_{58-66}$	9-mer	2*04:01	-	
RhoC56	YDRLRPLSY	$RhoC_{66-74}$	9-mer	3*04:01	-	
RhoC57	YDRLRPLSYPD	$RhoC_{66-76}$	11-mer	3*04:01	-	
RhoC58	YPDTDVILM	$RhoC_{74-82}$	9-mer	1*07:02	1.2	x
RhoC59	YPDTDVILMCF	$RhoC_{74-84}$	11-mer	1*04:01<br>1*07:02	1.0<br>0.8	N/A
RhoC60	YVPTVFENY	$RhoC_{34-42}$	9-mer	1*07:02<br>2*04:01	-<br>-	
RhoC61	YVPTVFENYI	$RhoC_{34-43}$	10-mer	2*05:02	0.7	
RhoC62	AAIRKKLVI	$RhoC_{2-10}$	9-mer	2*05:02<br>3*04:01	N/A<br>N/A	

Summarized table for the 198 9-11-mer peptide library. Peptides are listed according to name (first column) with indication of aa sequence (second column), position in the protein (third column), peptide length (fourth column) and SLA-restriction according to *in silico* analysis (fifth column). Determination of the actual binding to a given SLA-molecule as determined by the SPA analysis is listed in the sixth column, where $t_{1/2}$ (hours) is listed for each of the stably binding peptides ($t_{1/2} \geq$ 0.5 h). The SPA analysis was unsuccessful for a few peptides as indicated by N/A. For the T cell responses, “x” indicates that a response was found to this given peptide at least once, and SLA restriction is not included here. A response is defined as a 2-fold increase as compared to day -2 and at least 25 pg/ml of IFN-γ. N/A for T cell responses indicates that the PBMCs were not co-cultured with this peptide due to difficulties in the SPA analysis thereby delaying determination of binding stability for a few peptides.
